# The Relationship Between Electrical Energy Delivered by Deep Brain Stimulation and Levodopa-Induced Dyskinesias in Parkinson's Disease: A Retrospective Preliminary Analysis

**DOI:** 10.3389/fneur.2021.643841

**Published:** 2021-05-31

**Authors:** Marco Prenassi, Mattia Arlotti, Linda Borellini, Tommaso Bocci, Filippo Cogiamanian, Marco Locatelli, Paolo Rampini, Sergio Barbieri, Alberto Priori, Sara Marceglia

**Affiliations:** ^1^Dipartimento di Ingegneria e Architettura, Università Degli Studi di Trieste, Trieste, Italy; ^2^Newronika SpA, Milan, Italy; ^3^Fondazione Istituto di Ricovero e Cura a Carattere Scientifico (IRCCS) Ca'Granda Ospedale Maggiore Policlinico, Milan, Italy; ^4^“Aldo Ravelli” Research Center for Neurotechnology and Experimental Brain Therapeutics, University of Milan Medical School, Milan, Italy; ^5^Dipartimento di Fisiopatologia Medico-Chirurgica e dei Trapianti, Università degli Studi di Milano, Milan, Italy

**Keywords:** total electrica energy delivered, adaptive deep brain stimulation, dyskinesia, local field potential (LFP), safety

## Abstract

**Background:** Adaptive Deep Brain Stimulation (aDBS) is now considered as a new feasible and effective paradigm to deliver DBS to patients with Parkinson's disease (PD) in such a way that not only stimulation is personalized and finely tuned to the instantaneous patient's state, but also motor improvement is obtained with a lower amount of energy transferred to the tissue. Amplitude-controlled aDBS was shown to significantly decrease the amplitude-driven total electrical energy delivered to the tissue (aTEED), an objective measure of the amount of energy transferred by DBS amplitude to the patient's brain. However, there is no direct evidence of a relationship between aTEED and the occurrence of DBS-related adverse events in humans.

**Objective:** In this work, we investigated the correlation of aTEED with the occurrence of levodopa-induced dyskinesias pooling all the data available from our previous experiments using aDBS and cDBS.

**Methods:** We retrospectively analyzed data coming from 19 patients with PD undergoing surgery for STN-DBS electrode positioning and participating to experiments involving cDBS and aDBS delivery. Patients were all studied some days after the surgery (acute setting). The aTEED and dyskinesia assessments (Rush Dyskinesia Rating Scale, RDRS) considered in the Med ON-Stim ON condition.

**Results:** We confirmed both that aTEED values and RDRS were significantly lower in the aDBS than in cDBS sessions (aTEED mean value, cDBS: 0.0278 ± 0.0011 j, vs. aDBS: 0.0071 ± 0.0003 j, *p* < 0.0001 Wilcoxon's rank sum; normalized RDRS mean score, cDBS: 0.66 ± 0.017 vs. aDBS: 0.45 ± 0.01, *p* = 0.025, Wilcoxon's rank sum test). In addition, we found a direct significant correlation between aTEED and RDRS (ρ = 0.44, *p* = 0.0032, Spearman's correlation).

**Conclusions:** Our results provide a first piece of evidence that aTEED is correlated to the amount of levodopa-induced dyskinesias in patients with PD undergoing STN-DBS, thus supporting the role of aDBS as feasible and safe alternative to cDBS.

## Introduction

Increasing evidence supports the safety, feasibility, and efficacy of new deep brain stimulation (DBS) strategies that implement closed-loop adaptive stimulation for patients with Parkinson's Disease (PD) implanted in the subthalamic nucleus (STN) (1–8). Adaptive DBS (aDBS) differs from conventional DBS (cDBS) because it provides electrical stimulation with parameters that are changed real-time according to the analysis of brain signals, especially local field potentials (LFPs) recorded from the same lead implanted for DBS (9, 10). Conversely, cDBS provides a fixed-parameters stimulation, in which amplitude, frequency, and pulse-width remain unchanged throughout the day, without following the clinical state of the patient. The aDBS concept is based on a simple closed-loop model in which a feedback variable is sensed by the system in order to assess the patient's state, and a control algorithm provides the new stimulation parameters to be delivered to the patient (9). To date, even though several options were explored, including external sensors and electrochemical biomarkers (10, 11), there is general consensus on the use of STN LFP-based biomarkers to represent the clinical state of the patient. Among possible oscillations, the LFP beta band (13–35 Hz) has been targeted in most of the experiments in humans (1, 4, 12). There are however other frequency bands suitable to control specific symptoms, such as the gamma (8, 11), or low-frequencies (13), or a combination of them (14). Among possible stimulation parameters (namely amplitude, frequency, and pulse width), all the present implementations and proof-of-concepts of aDBS are based on amplitude-control, whereas frequency and pulse width are kept constant. In fact, even though different frequencies (15, 16) and shorter pulse widths (17, 18) were studied in PD patients, the complexity behind frequency- and pulse width-modulations is still too high to be successfully implemented in closed-loop algorithms.

The general hypothesis behind amplitude-controlled aDBS is to provide an effective stimulation, while reducing the amount of unnecessary stimulation that is delivered by cDBS (19, 20). Stimulation-related side effects are known in patients treated with DBS and are usually reversed by reprogramming (21–24) or by switching stimulation OFF (23). However, in the long term, some of these reversible effects become non-reversible (23, 24), and tend to worsen. This could be due to disease progression, alterations in postoperative medications, comorbidities, but also to possible long-term effects of stimulation, as hypothesized for worsened axial symptoms (25). In a similar way, in essential tremor, patients are instructed, if possible, to switch off DBS during the night, in order to decrease the “tolerance effect” (i.e., the need to increase stimulation amplitude to maintain tremor control) (26). In addition, DBS has been shown to impair response inhibition (27), possibly leading to stimulation-induced impulsivity (19, 20, 28, 29) that could result from excessive beta suppression produced by continuous stimulation (20).

Amplitude-controlled aDBS was shown to significantly decrease the amount of energy delivered to the tissue, measured through a specific quantitative parameter (the total electrical energy delivered to the tissue, TEED) (30). Since the present aDBS applications keep frequency and pulse width fixed, the TEED calculated in aDBS experiments is an amplitude-driven TEED (aTEED). In aDBS, decreasing the energy delivered to the tissue is not necessarily related to battery saving, because there is a power consumption related to the sensing and control circuits that counterbalances the saving in stimulation output. This depends also on the type of adaptation strategy implemented. For instance, there are types of aDBS specifically designed to decrease the stimulation delivered to the tissue by switching DBS ON and OFF (1), whereas other types of aDBS are designed to linearly follow the beta power, continuously changing stimulation amplitude within a stimulation window, without switching DBS OFF at any time (4, 31).

The present STN-LFP aDBS implementations follow the dynamic of beta oscillation that is correlated with bradykinesia and rigidity, and that is highly influenced by levodopa dynamics. It is therefore intuitive that an automatic adaptation of stimulation amplitude following beta changes can help controlling the summative effects of medication and stimulation, especially on dyskinesia (21, 32).

Since implantable devices delivering aDBS are not yet on the market, the effects of aDBS were mostly studied in the acute setting, immediately after the surgery for DBS electrode implant. In this limited experimental setting and with the limits of the present implementation that is based on a single biomarker (beta-band LFP), the literature shows that aDBS controls motor symptoms at least as well as cDBS, and sometimes even better than cDBS, and, in addition, provides better control of levodopa-induced dyskinesias when DBS is ON (4–6) and speech disturbances (3), which are common DBS-related side effects.

Even though the acute experimental setting is not suitable for driving definite conclusions on the effectiveness and on the optimal approach of aDBS especially in the long term, it is however possible to start assessing the relationship between aTEED and DBS-related adverse events that occur in the short-term, such as levodopa-induced dyskinesia.

In order to provide an initial piece of evidence on the role of aTEED in the occurrence of stimulation related side effects, in this work, we investigated the correlation of aTEED with levodopa-induced dyskinesias retrospectively considering all the data available from our previous experiments using aDBS and cDBS (5, 6).

## Methods

This is a retrospective study involving 19 rigid-akinetic patients with advanced PD who underwent surgery for a subthalamic nucleus DBS electrode implantation in the Neurosurgery Unit at Fondazione IRCCS Ca'Granda Ospedale Maggiore Policlinico Hospital in Milan. No surgical complication was experienced by these subjects. Subjects were involved in three studies: the first one was reported by Rosa et al. (5), the second one by Arlotti et al. (6), and the third one is currently in press. All studies were approved by the institutional review board of the Policlinico Hospital and conformed with the Declaration of Helsinki. All patients provided written informed consent to the experimental procedures. Of the 19 patients here reported, 6 were included in the first study (5), 8 in the second one (6), and 5 in the third study. The patients were included in this study if all the measures and scales described below were available. All the experimental sessions took place few days after DBS surgery before the leads were connected to the subcutaneous pulse generator.

### Experimental Procedure

**Figure 1:**
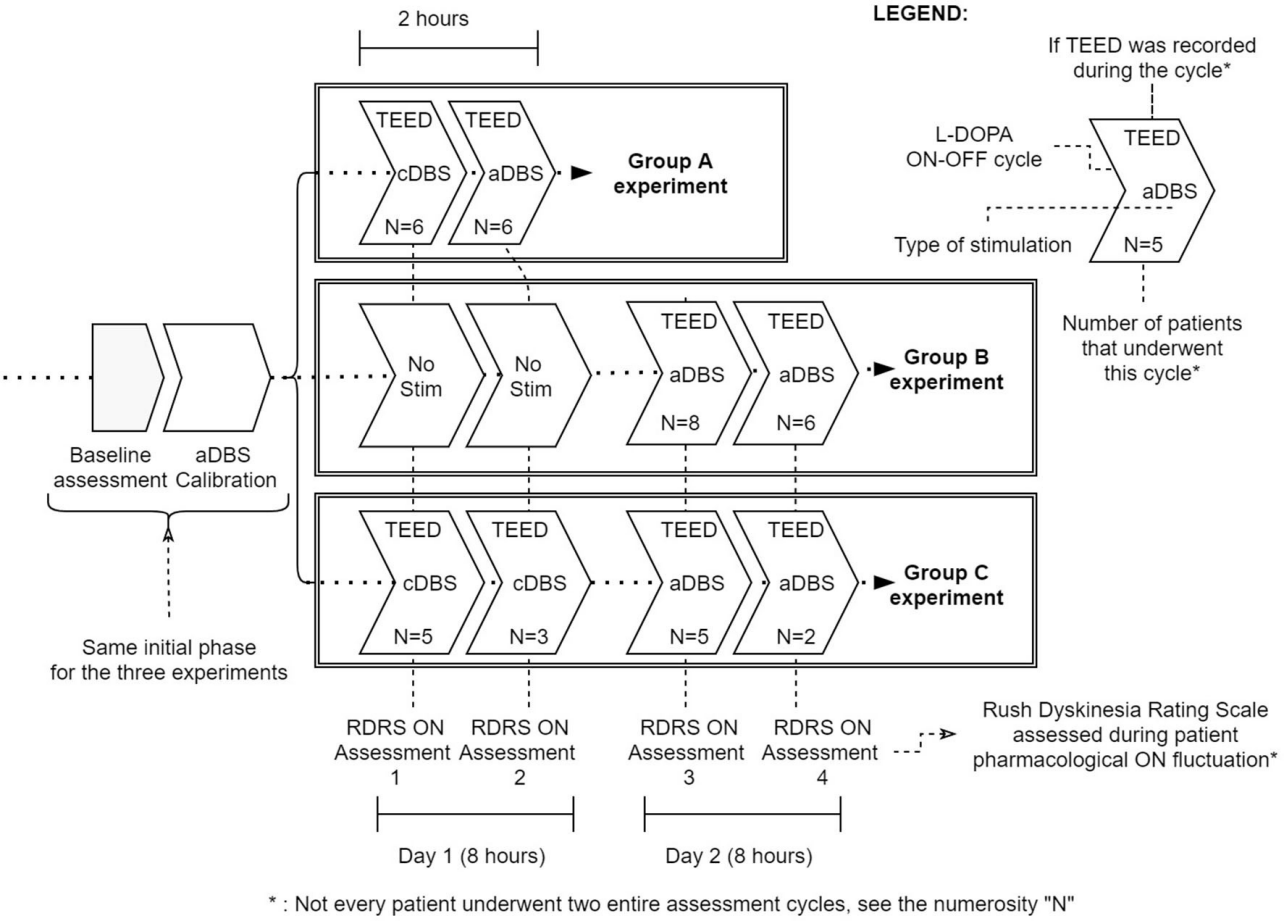
Summary of the three experimental protocols (Group A, Group B, and Group C). Baseline assessment [including Unified Parkinson's Disease Rating Scale part III and Rush Dyskinesia Rating Scale (RDRS) evaluation] and aDBS calibration are common to all protocols. Then, Group A underwent a 2-h cDBS or aDBS exposure (randomized) with a full levodopa cycle per day. Group B and C were studied for two levodopa cycles per day (8 h study). In Group B patients during day 1 received only levodopa while in day 2 received aDBS; in Group C patients received cDBS in day 1 and aDBS in day 2 (not randomized). In each levodopa cycle we considered TEED and RDRS in Med ON - Stim ON. N represents the number of subjects assessed in each cycle.

The experimental procedures were fully detailed in the previous studies (5, 6), and are summarized in Figure 1.

In brief, the patients from the first study were recorded, evaluated, and stimulated in two different sessions of 2 h each; in one session was delivered aDBS and in the other one cDBS in random order (5). Each experimental session lasted at least 2 h, during which the patient was evaluated three times: (1) after a baseline assessment (OFF DBS and OFF medication, stimOFF/medOFF, after a night withdrawal of medication), (2) after the stimulation was turned on and before levodopa administration (stimON/medOFF, at least 30 min after DBS was turned on), and (3) 40 min after the administration of the usual patient's levodopa dose increased by 50% (stimON/medON condition). Clinical assessments included an evaluation of motor performance, and dyskinesias.

The patients from the second study were recorded and evaluated in two different days (day 5 and 6 from the electrode implantation), in two different sessions of 8 h each. On the first session, the subjects were recorded and evaluated with normal levodopa intake but without stimulation. On the second session they were stimulated with aDBS; recording, levodopa intake and assessment parameters were the same as of the day before. The patients, in both days, were assessed and recorded in ecological settings (during hospitalization) and freely moving (6).

The patients from the third study were assessed following the same protocol as in Arlotti et al. (6) with the exception that on the first session they received cDBS instead of no stimulation. The day after, they received aDBS.

All the clinical details of the patients of the three groups are reported in Table 1.

**Table 1:** Patient's clinical characteristics.

						**Preoperative assessment^‡^**		**Stimulation parameters**				
**Case**	**Sex**	**Age at surgery**	**Disease duration [years]**	**Onset side**	**Preoperative LEED [mg]**	**UPDRS III score, medication “off”**	**UPDRS III score, medication “on”**	**Voltage cDBS [V]**	**Freq. [Hz]**	**Width [μs]**	**Voltage Range aDBS [V]**	**Reference study**
A1	M	54	19	R	479	26	8	2	130	60	0–2	(5)
A2	M	50	15	R	892	37	14	2	130	60	0–2	
A3	M	48	7	L	1,019	43	27	2	130	60	0–2	
A4	F	68	16	R	1,648	23	8	2	130	60	0–2	
A5	M	59	9	L	1,200	30	5	2	130	60	0–2	
A6	M	51	8	L	975	20	6	2	130	60	0–2	
B1	M	59	10	R	208	25	13	3.5	130	60	0.1–3.5	(6)
B2	M	62	9	R	686	41	18	4	130	60	0.1–4	
B3	M	67	12	R	1,494	25	10	3	130	60	0.1–3	
B4	M	50	8	R	1,055	32	16	3	130	60	0.1–3	
B5	M	47	11	R	1,080	19	10	3.5	130	60	0.1–3.5	
B6	M	70	12	L	872	13	3	2.5	130	60	0.1–2.5	
B7	M	54	13	L	785	24	8	3	130	60	0.1–3	
B8^*^	F	69	17	L	935	//	//	2	130	60	0.1–2	
C1	M	64	10	L	1,290	18	11	2.5	130	60	0.1–2.5	In press
C2	F	51	11	L	1,480	20	4	3.5	130	60	0.1–3.5	
C3	M	61	15	L	875	40	25	2.5	130	60	0.1–2.5	
C4^*^	M	70	19	L	1,280	//	//	3	130	60	0.1–3.0	
C5	F	49	9	R	793	37	14	2.5	130	60	1.5–2.5	
*N* tot.: 19	79% M 21% F	58 ± 8	12 ± 4	47% R 53% L	1,002 ± 353	28 ± 9	12 ± 7	2.5 ± 1	130	60	0.14 ± 0.33 2.7 ± 0.67	

*LEED, levodopa equivalent daily dose; UPDRS, Unified Parkinson's Disease Rating Scale*.

* *Case B8 and C4 preoperative UPDRS III score data is missing*.

‡ *Preoperative response to levodopa refers to UPDRS III score assessed by a neurologist at the time of indication for DBS surgery (≈6 months before surgery)*.

### aDBS and cDBS

In all studies, aDBS and cDBS were both delivered using the AlphaDBS V-ext system (31), an external portable device able to record and simultaneously deliver voltage-controlled DBS. The device has two modes: cDBS, in which the stimulation is delivered with fixed parameters, at the effective stimulation amplitude, and aDBS in which stimulation parameters are changed linearly according to the beta band power, with the stimulation amplitude ranging from 0 to the effective stimulation amplitude. AlphaDBS V-ext was calibrated the day before the first experimental session, according to the procedures described in Rosa et al. (4), in order to provide a personalized aDBS algorithm. aDBS and cDBS were delivered unilaterally, as described in Rosa et al. (5) and Arlotti et al. (6).

In all experiments, cDBS was delivered at 130 Hz, with 60 uV pulse width, and an amplitude that was set by an experienced neurologist according to the observed therapeutic window. cDBS amplitude for all patients receiving cDBS is reported in Table 1 and was kept constant throughout the experimental session (2 or 8 h).

aDBS was delivered according to the adaptive approach described in Arlotti et al. (31). In summary, the adaptive algorithm works only by changing DBS amplitude (pulse width is kept fixed at 60 us and frequency at 130 Hz). DBS amplitude is linearly changed in a pre-defined range (Vmin–Vmax) and it is never turned OFF. The amplitude change follows the beta band amplitude changes as recorded from DBS electrodes. aDBS amplitude ranges (Vmin–Vmax) for all patients are reported in Table 1.

### Data Analysis

In this work, we considered dyskinesia assessments conducted using the Rush Dyskinesia Rating Scale (RDRS,cit.) which was available in all studies.

In order to pool data from different studies, we considered one clinical evaluation per patient performed when the medication was effective (peak dose = 45–60 min after levodopa administration) and the stimulation was active (Stim ON - Med ON), considering a timeframe of 20 min (±10 min) centered at the start of the clinical evaluation. The aTEED was calculated in the same time frame.

If the patient received both aDBS and cDBS, we considered one clinical evaluation and related aTEED per each type of stimulation.

The aTEED was calculated using equation 1 following the procedures described in Koss et al. (30). The impedance was measured with a impedance meter (Model EZM 4; Grass, USA) at 30 Hz.

(1) $$\begin{array}{r} TEED=\overset{N}{\underset{n=0}{\sum }}\frac{{V\left(nT\right)}^{2}p_{w}f}{Z_{r}}\;\left[J\right] \end{array}$$

*V*(*nT*): stimulation voltage at the time instant nT [V] – note that in cDBS this value is fixed whereas for aDBS it changes every T (1 s);

*T* time period, fixed at 1 s for every patient [s];

*N*: 20 min timeframe, equal to 1,200 samples for every patient (1 sample/s);

*p*_*w*_: pulse width [s] = 6^*^10(-6) s;

*f*: frequency [Hz] = 130 Hz;

*Z*_*r*_: Impedance module at 30 Hz, sinusoidal wave [Ω], referred to the stimulating contact.

### Statistical Analysis

Since the purpose of this study is to verify whether the amount of stimulation could be related to levodopa induced dyskinesias, and since aTEED depends only on the instantaneous DBS amplitude (pulse width and frequency are always fixed at 130 Hz and 60 us) each pair (aTEED, RDRS) was considered as an independent sample, whatever the type of stimulation.

However, to account for the heterogeneity of patients (i.e., the amount of peak dyskinesia differs among patients, as well as the optimal cDBS voltage), we scaled each RDRS and aTEED value by the maximum value of RDRD and aTEED registered in each study protocol. Therefore, the RDRS and aTEED values of patients coming from Rosa et al. (5) were divided by 8 and 30 mJ, respectively, those coming from the (6) study by 18 and 22.8 mJ, and those from the third study in press by 8 and 52.3 mJ. This normalization methodology was chosen in order to preserve the exact scale between the data even though the ranges differ among studies. Then, non-parametric statistics was applied.

As a first step, in order to allow pooling all the data together, we verified that, after normalization, no significant differences were observable between patients belonging to the three studies for both aDBS and cDBS (Kruskal-Wallis test, *p* > 0.025 as per Bonferroni correction).

Second, we verified that, according to the literature, aTEED values were lower in aDBS than in cDBS, and that aDBS was able to better control dyskinesia than cDBS. Since not all patients received both aDBS and cDBS, we considered the two groups as independent, even though some patients received both aDBS and cDBS in two different sessions. To allow the use of independent statistics we verified that there was no correlation between aDBS and cDBS in those patients receiving both treatments (aTEED: *p* = 0.2, RDRS: *p* = 0.2). Then, we compared the measures of aTEED and RDRS in aDBS (*N* = 27, see Figure 1) vs. cDBS (*N* = 14, see Figure 1), using non-parametric statistics (Wilcoxon's rank sum test, *p* < 0.05).

Then, we investigated whether there was a correlation between aTEED and RDRS values, using Linear Regression Analysis conducted using the Spearman's coefficient, since the cohort considered in this study is small (*p* < 0.05). For the correlation analysis, we considered all data available for each patient (all evaluations in the Med On-Stim ON condition, both with aDBS and cDBS), for a total of *N* = 41 samples.

Throughout the text, all data are expressed as [mean ± standard error]. All analyses were carried out using the software MATLAB Version 9.3.0.713579 (R2017b, TheMathWorks Inc.,USA).

## Results

Table 2 reports the means and standard error for aTEED and RDRS in each study protocol. No significant differences were observed neither in the normalized energy delivered (cDBS sessions *p* = 0.91, aDBS sessions *p* = 0.15) nor in the normalized dyskinesia scores (cDBS sessions *p* = 0.07, aDBS sessions *p* = 0.08), thus allowing to pool the data from these studies together.

**Table 2:** Patient's clinical assessments.

**Group**	**aTEED cDBS**	**Normalized**	**aTEED aDBS**	**Normalized**	**RDRS**	**Normalized**	**RDRS**	**Normalized**
	**[mJ]**	**aTEED cDBS**	**[mJ]**	**aTEED aDBS**	**cDBS**	**RDRS cDBS**	**aDBS**	**RDRS aDBS**
A	22 ± 1.2	0.73 ± 0.042	6.4 ± 1.1	0.21 ± 0.038	5.7 ± 0.34	0.71 ± 0.043	5.17 ± 0.41	0.65 ± 0.052
B^*^	//	//	2.7 ± 0.2	0.22 ± 0.017	//	//	6 ± 0.32	0.33 ± 0.018
C	36.5 ± 2.9	0.70 ± 0.055	16.4 ± 1.1	0.31 ± 0.021	4 ± 0.27	0.5 ± 0.034	2.5 ± 0.18	0.31 ± 0.023
Total Mean ± st.dev	28.6 ± 2.1	0.72 ± 0.047	10.95 ± 1.05	0.24 ± 0.033	4.9 ± 0.29	0.61 ± 0.037	4.82 ± 0.41	0.43 ± 0.041

*The three groups refer to A: (5); B: (6); C: in press*.

* *B group protocol does not involve cDBS stimulation*.

*st.dev., standard deviation*.

As expected, we found that aTEED values were significantly lower in the aDBS than cDBS sessions (aTEED mean value, cDBS: 0.0278 ± 0.0011 j, vs. aDBS: 0.0071 ± 0.0003 j, *p* < 0.00001, Wilcoxon's rank sum).

We also confirmed previous findings showing that aDBS is able to better control dyskinesias when the patients are in the ON medication state with DBS ON. The normalized RDRS assessed during both sessions was significantly lower during the aDBS than cDBS (normalized RDRS mean score, cDBS: 0.66 ± 0.017 vs. aDBS: 0.45 ± 0.01, *p* = 0.025, Wilcoxon's rank sum test).

**Figure 2:**
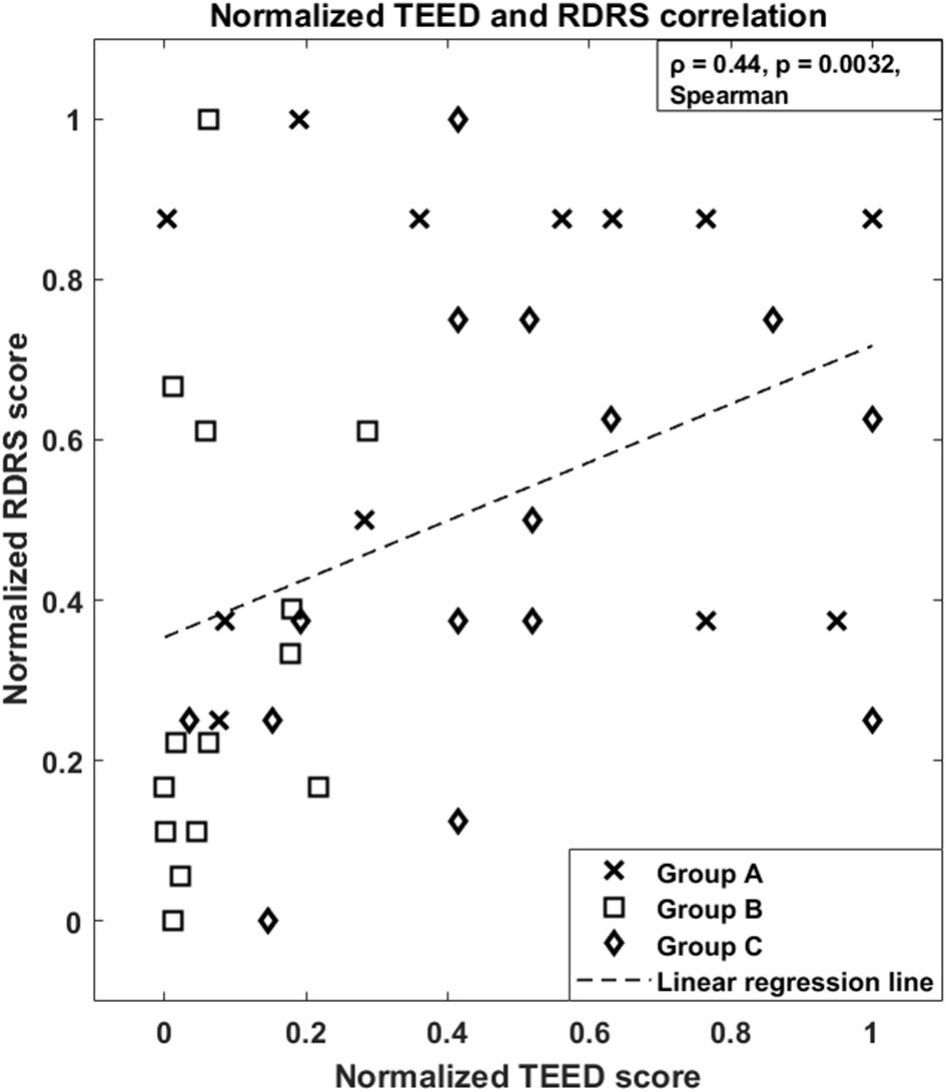
Normalized Total Electrical Energy Delivered to the tissue, aTEED, and normalized Rush Dyskinesia Rating Scale, RDRS, correlation of the three studies. Group A: (5), *N* = 12; Group B: (6), *N* = 14; Group C: in press, *N* = 15 (total *N* = 41). The dashed line represents the estimated linear regression.

Finally, Figure 2 shows that there is a direct relationship between the amount of energy delivered by DBS and the amount of dyskinesias observed in each patient (ρ = 0.44, *p* = 0.0032, Spearman's correlation).

## Discussion

In this work, retrospectively evaluating the energy delivered to the tissue in LFP-based aDBS and cDBS, together with the amount of dyskinesia experienced by the patients in the ON medication-ON stimulation state, we provided, for the first time, a preliminary piece of evidence that stimulation-induced side effects are correlated with the electrical energy delivered to the tissue by DBS amplitude.

Even though aTEED reduction has been previously considered beneficial in several clinical trials on neurostimulation systems (33, 34) as a measure of safety, no direct evidence of a relationship between aTEED and the occurrence of adverse events was available in humans so far. Our results therefore support the possibility to use this parameter, which is an objective quantitative measure that can be provided directly by implanted devices, as a safety measure in future trials involving amplitude-controlled DBS.

In fact, in aDBS, it is possible to take advantage of the whole therapeutic window allowed by DBS (from the minimum effective amplitude to the maximum amplitude before the occurrence of stimulation-related side effects). This is not possible with cDBS because it is necessary to define a compromise between efficacy and long-term stimulation with fixed parameters (21). Therefore, aDBS can reach, for short time windows, amplitudes higher than cDBS, keeping the aTEED in any case lower than cDBS.

It is important to underline that a lower aTEED does not imply a lower battery consumption, at least in current aDBS technological implementations. In fact, the pulse generator has to include a sensing technology allowing biopotential recordings when DBS is turned ON (10) and this circuit consumes energy, possibly counterbalancing the benefits of lower stimulation. In addition, the use of aTEED has to consider a methodological limitation. The relation we found between energy delivered and neurophysiological/clinical effects cannot be considered monotonical or linear in general, but only in case of DBS with fixed pulse width and frequency. When more complex algorithms, involving the modulation of pulse width and/or frequency, will be implemented in aDBS systems, the linearity of the relationship would be lost and more complex mechanisms will be involved. Therefore, the role of TEED should be re-evaluated, and the use of aTEED would be not appropriate.

Even though evaluated retrospectively, these results seem to confirm, in a larger sample of patients, that LFP-based aDBS better controls levodopa-induced dyskinesias in the ON medication-ON stimulation state, as already reported (5, 6). Previous studies have recently shown a significant inverse correlation between subthalamic neuronal firing and total electrical energy delivered (TEED), with higher TEED leading to an increased suppression of neuronal firing (35). Given the overall inhibitory role exerted by the subthalamus on the indirect basal ganglia pathway, we can hypothesize that lower aTEED leads to milder dyskinesias.

This work has however some limitations that should be taken into account when interpreting the results. First, in the study we pooled together the results of aDBS and cDBS in patients undergoing different protocols. The variability is mainly related to the total time of aDBS/cDBS delivery to the patients, being those coming from the first protocol studied for 2 h, while the others for 8 h. However, this variability is lowered by several aspects: the device (AlphaDBS-V-ext) is the same for all the studies; patients were all predominantly rigid-akinetic and were operated by the same surgical team, thus lowering the probability to introduce biases related to electrode positioning; the time frame considered to study the correlation between aTEED and dyskinesias is the same for all patients (20 min around the levodopa peak). Another limitation is that all studies were conducted in the acute setting, some days after the surgery for electrode placement, in a condition that is intrinsically biased by the microlesioning effect of lead placement, as well as by the possible presence of edema. In particular, regarding the aTEED calculation, the impedance in this specific time frame is lower than in the chronic setting (36), thus lowering the possibility to generalize our results. However, the impedance change over time should be consistent across subjects, thus limiting the effect on the correlation. Another point is that RDRS, while being an easy tool, focuses on functional ability, and does not consider the body spreading or duration of dyskinesia. Finally, we cannot rule out the possibility that the observed correlation is specific of the sample studied. This may be due to the fact that all patients received DBS implant in the same institution, thus limiting the effects of variable lead positioning due to heterogeneous procedures, and underwent the same type of aDBS. However, we are not proposing a regression model to establish a direct relationship between aTEED and dyskinesia scores, but we only verified the existence of a correlation. All these limitations can be mitigated designing a larger and prospective study, which should be able to confirm the hypothesis that the amount of energy delivered to the tissue in an amplitude-controlled approach is related to the occurrence of dyskinesias.

The present aDBS implementation, based on a single biomarker (beta band LFP) is limited by the fact that not all patients treated with DBS show a consistent beta peak (37), and, therefore, patients without significant beta activity were excluded from all the studies on closed-loop DBS (1, 5). This first biomarker was chosen because of its well-known and recognizable dynamic that made it as a suitable candidate for a first implementation. However, aDBS should be considered as a platform, in its very early stages, that will in the future provide personalized DBS therapy, with biomarkers specifically targeted on patient's symptoms. The present preliminary results, together with the first implantable commercial devices (Medtronic Percept^TM^, Newronika AlphaDBS System) with sensing capabilities currently under industry-sponsored clinical trials (i.e., NCT04547712 and NCT04681534), represent the starting point for such patient's tailored DBS therapy.

In conclusion, our results provide a first piece of evidence that aTEED is correlated to the amount of levodopa-induced dyskinesias in patients with PD undergoing STN-DBS, with electrophysiological characteristics suitable for beta band LFP-based aDBS, thus supporting the role of this type of aDBS as feasible and safe alternative to cDBS.

## Data Availability Statement

The raw data supporting the conclusions of this article will be made available by the authors, without undue reservation.

## Ethics Statement

The studies involving human participants were reviewed and approved by Ethical Committee of the Fondazione IRCCS Ca' Granda Ospedale Maggiore Policlinico of Milan. The patients/participants provided their written informed consent to participate in this study.

## Author Contributions

SM and MP conceived the idea, participated to the experiments, conducted data analysis, drafted the manuscript and the figures, and contributed to the design of the work and substantial revision of the work. TB, LB, and FC contributed in the design of the work and acquisition of data. PR and ML conducted the implantation surgery and contributed to data interpretation. All authors have approved the submitted version.

## Conflict of Interest

SM, AP, SB, FC, PR, and ML are founder and shareholder of Newronika SpA, a spin-off company of the Fondazione IRCCS Ca' Granda Ospedale Maggiore Policlinico and of the University of Milan. MA was employed by the company Newronika SpA. The remaining authors declare that the research was conducted in the absence of any commercial or financial relationships that could be construed as a potential conflict of interest.

## Abbreviations

DBS: Deep Brain Stimulation
LFP: Local Field Potentials
PD: Parkinson's Disease
RDRS: Rush Dyskinesia Rating Scale
TEED: Total Electrical Energy Delivered to the tissue.
